# Case report: Lysine improvement in siblings with glutaric acidemia type 1 following reduced medical food intake: Implications for amino acid absorption and reabsorption

**DOI:** 10.1016/j.ymgmr.2025.101262

**Published:** 2025-09-29

**Authors:** Grace Noh, Jariya Upadia

**Affiliations:** a Hayward Genetics Center, USA; b Department of Pediatrics, Tulane University School of Medicine, 1430 Tulane Ave SL-31, New Orleans, LA, USA

**Keywords:** Glutaric acidemia type 1, Arginine-fortified formula, Lysine-free formula, Cationic amino acid transporters, Lysine absorption, Lysine reabsorption, Dietary protein intake

## Abstract

Glutaric acidemia type 1 (GA1) is a rare metabolic disorder requiring dietary management with lysine (Lys)-free, tryptophan (Trp)-reduced, and arginine (Arg)-fortified medical formulas. This case report describes three siblings with GA1 who exhibited persistently low plasma lysine levels despite meeting age-appropriate dietary protein intake from intact protein sources. Upon reducing medical formula intake while maintaining intact protein consumption, lysine levels normalized, suggesting that excess dietary arginine may inhibit lysine intestinal uptake or reabsorption in the kidneys. These findings highlight the need for further research on amino acid interactions in GA1 dietary management and emphasize careful formula prescription to prevent unintended lysine deficiency.

## Graphical abstract

**Figure:**
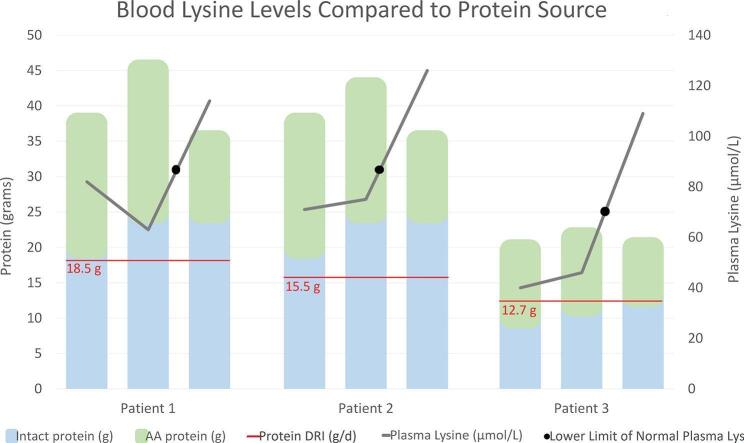

## 1 Introduction

Glutaric acidemia type 1 (GA1, OMIM#231670) is an autosomal recessive disorder caused by a deficiency of mitochondrial flavin-dependent glutaryl-CoA dehydrogenase (GCDH, EC 1.3.8.6), leading to impaired metabolism of lysine, tryptophan, and L-hydroxylysine [1]. As a result of GCDH deficiency, there is a buildup of glutaric acid, 3-hydroxyglutaric acid, and glutarylcarnitine in bodily fluid and tissues, including the blood, urine, cerebrospinal fluid, and brain tissue [1,2]. Patients with GA1 may present with congenital or infantile-onset macrocephaly while remaining otherwise asymptomatic. However, primarily between 6 and 18 months of age, they are at risk of experiencing an encephalopathic crisis often triggered by a febrile illness, fasting, or another catabolic state. This can lead to striatal damage and associated psychomotor issues [3,4].

Early intervention includes dietary restriction of lysine and tryptophan in patients up to 6 years of age, along with lifelong oral carnitine supplementation and intermittent emergency treatment during acute illness or other catabolic states. Dietary management also involves supplementing with a lysine (Lys)-free, tryptophan (Trp)-reduced, and arginine (Arg)-fortified medical formula to provide adequate vitamins, minerals, calories, and non-offensive amino acids [5,6]. With the implementation of expanded newborn screening (NBS), patients with GA1 are experiencing improved outcomes [3,7]. However, despite these advancements, efforts to optimize dietary management are ongoing, particularly in understanding the effects of lysine-free, arginine-fortified formulas [8].

Medical food designed for GA1 treatment is lysine-free, low in tryptophan, and fortified with high amounts of arginine. This composition aims to limit cerebral lysine uptake as arginine and lysine compete for the same blood-brain barrier facilitative transporter, high affinity cationic amino acid transporter-1 (CAT-1/SLC7A1) [9,10]. Arginine and lysine, both basic amino acids, compete for absorption through a sodium-dependent amino acid transporter in the intestinal luminal membrane and renal proximal tubule [[11], [12], [13]]. The impact of arginine on gut absorption of lysine and reabsorption in the kidneys and its impact on plasma amino acids levels in GA1 treatment remains less understood.

We observed improvement of plasma lysine levels in siblings with GA1, whose levels had previously been chronically low, following reduction of Lys-free, Trp-reduced, and Arg-fortified formula, independent of intact protein intake. This observation raises important questions about the interaction between arginine and lysine in dietary management and underscores the need for further research into the role of arginine-fortified medical foods in modulating plasma amino acid levels. These findings raise important questions regarding the competitive interaction between arginine and lysine and its clinical significance in dietary management.

## 2 Case report

Three siblings, currently 6 years (male), 5 years (female), and 3 years (male), were initially presumed positive for GA1 based on their newborn screening results (MS/MS). The reports revealed elevated C5DC_C6OH of 0.87 μmol/L, 0.82 μmol/L, and 1.21 μmol/L (cut-off reference: >0.5 μmol/L), respectively. Additionally, elevated C5DC/C16 ratios were reported in the younger siblings at 0.40 μmol/L and 0.37 μmol/L (cut-off reference: >0.25 μmol/L). Trace to moderate amounts of glutaric acid and 3-hydroxyglutaric acid were found in their urine. The diagnosis of GA1 was confirmed by the identification of a homozygous pathogenic variant, C.572 T > C (p.Met191Thr), in the *GCDH* for all three siblings. Upon diagnosis, the patients were promptly referred to a biochemical geneticist in New Orleans. They were born at term, with an uncomplicated prenatal and neonatal course. Growth parameters for weight, height, and head circumference were within normal limits for all siblings. There is no known family history of GA1, striatum damage, or psychomotor issues and parents are non-consanguineous.

Lys-free, Trp-reduced, and Arg-fortified formula was initiated at three months of age for the oldest sibling and within the first week of life for the younger two. In the oldest sibling, medical formula initiation was delayed due to exclusive breastfeeding, which initially met lysine intake requirements (65–100 mg lysine/kg/day from 0 to 6 months of age) [13]. Medical food was added once well baby formula was needed. Carnitine supplementation was started at 50 mg/kg/day and adjusted as needed based on weight gain for all siblings. Upon the introduction of solid foods, parents were provided with a daily protein goal and instructed to track protein consumption from dietary sources. All three siblings have met developmental milestones, and none have experienced striatal injury to date.

During the first six months of life, lysine intake ranged between 70 and 100 mg/kg/day among the siblings. Intact protein sources, including breastmilk, well-baby formula, and/or foods, were gradually increased based on blood lysine levels. By early childhood, each child was consuming protein at levels consistent with the age-appropriate Dietary Reference Intake (DRI). However, despite meeting the DRI, all three siblings developed chronically low lysine levels. Lysine, tryptophan, arginine, and calorie intake were not quantifiable due to lack of dietary records. To monitor appropriate protein intake, we used the lab reference ranges for amino acids. Proposed guidelines suggest maintaining lysine and other essential amino acids within the normal range [6].

**Fig. 1:**
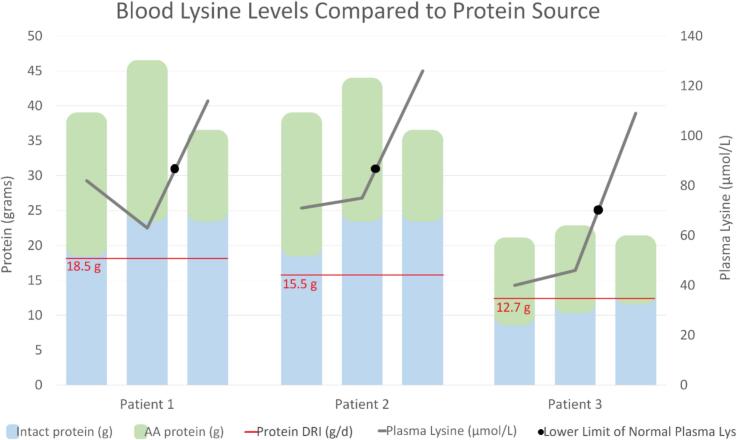
Trends in blood lysine levels (grey solid line) in relation to amino acid (AA)-based protein intake (source: Lys-free, Trp-reduced, Arg-fortified formula, green bar) and intact protein intake (blue bar) in three siblings with GA1. Despite increasing intact protein intake, lysine levels remained below the reference range (black dot indicates the lower limit of normal) when AA-based protein intake remained constant. Lysine levels normalized only after a reduction in AA-based protein intake, while intact protein intake remained stable.

Attempts to increase intact protein beyond the DRI by 30–60 % had no impact on chronically low blood lysine levels. However, when intake of Lys-free, Trp-reduced, and Arg-fortified formula was reduced by 30–50 % while maintaining intact protein intake, blood lysine levels normalized (Fig. 1). Notably, plasma Lys/Arg ratios were inversely related to formula intake, averaging 0.91 at the first collection, dropping to 0.62 after increased intact protein, and rising to 1.52 following reduced Lys-free, Trp-reduced, Arg-fortified formula. The timing of GA1 formula consumption and lysine intake varied among the siblings at the time of lab collections. The older siblings drank formula one to two times per day alongside lysine-containing foods, while the youngest sibling consumed a mixture of GA1 formula and standard infant formula throughout the day. While the family reported protein intake verbally, no detailed diet records or food frequency questionnaires were available for review. Due to the lack of quantifiable data on lysine and arginine content in foods, we are unable to determine exact values on lysine and arginine intake and their relationship to one another. A calorie modular was introduced to all three to help meet energy needs, and growth was monitored throughout these dietary adjustments. The family was advised to include one serving of a high-quality protein source daily to meet the DRI. Despite these limitations, the normalization of blood lysine levels coincided with the implementation of these dietary adjustments and efforts.

## 3 Discussion

To reduce the risk of metabolic decompensation and neurological complications in GA1, incorporating Lys-free, Trp-reduced, and Arg-fortified formula alongside a low-protein diet and careful management of intact protein intake is recommended. Increased arginine from this specialized formula has been shown to significantly suppress plasma lysine levels as observed in our study and others [14]. Findings from this report suggest that while Lys-free, Trp-reduced, Arg-fortified formula is commonly used in the treatment of GA1, it may provide excessive arginine, potentially contributing to persistently low lysine levels, even in patients who are meeting dietary lysine and tryptophan requirements. Notably, lysine levels normalized following a reduction in formula intake, despite no change in intact protein consumption. Growth was monitored throughout these dietary adjustments and remained within normal parameters, with no signs of malnutrition. The primary clinical finding was persistently low lysine levels, which may be important for other practitioners to consider when prescribing Lys-free, Trp-reduced, and Arg-fortified formula.

Lysine and arginine are both cationic amino acids and, as such, share common transport mechanisms across intestinal and renal epithelia. In the small intestine, their absorption is primarily mediated by the rBAT/b^0^,+AT transporter. Although high dietary levels of arginine may induce competitive inhibition and reduce lysine absorption, an alternative transporter, B^0^,+AT (*SLC6A14*), expressed in the distal regions of the intestine may facilitate lysine uptake, thereby partially mitigating the impact of competition. In the kidneys, lysine reabsorption may also be attenuated via competition with arginine for the same rBAT/b^0^,+AT transporter [13,15]. This mechanism was illustrated in a case report by Kato et al. (1982), in which a patient with lysinuric protein intolerance (LPI) and four healthy controls demonstrated reduced lysine reabsorption following intravenous arginine administration [16]. These findings suggest that further investigation into urinary lysine and arginine concentrations may provide additional insight into the effects of arginine supplementation in GA1 formulas.

The lack of detailed diet records and amino acid tracking limits the ability to draw definitive conclusions about the impact of arginine on lysine absorption and reabsorption. Although precise intakes of lysine, tryptophan, arginine, and other amino acids were not recorded, the family monitored daily protein intake. An international survey conducted by Bernstein et al. reported that most GA1 patients track grams of protein rather than milligrams of lysine [17]. While tracking protein intake is more practical due to the accessibility of nutritional data, this approach is less precise and does not account for the specific amino acid composition of foods. For instance, lysine is the first limiting amino acid in cereals and legumes [18], and diets restricted in protein tend to also be high in cereals. A diet that meets the DRI for protein but relies heavily on cereals as the primary protein source may result in insufficient lysine levels. To address this, incorporating at least one serving of a high-quality protein source daily may help ensure an adequate lysine supply.

Lysine is one of the nine essential amino acids that cannot be synthesized by the body and must be obtained through diet. It plays a crucial role in protein synthesis and serves as a precursor for carnitine biosynthesis [19]. A deficiency in essential amino acids, such as lysine, can contribute to overall protein deficiency, leading to reduced disease resistance, decreased blood protein levels, and impaired mental and physical development in young children [18].

In comparison, standard well-baby formulas typically contain 300–350 mg of arginine per 100 g, whereas arginine-fortified lysine-free infant formulas contain 1000–1500 mg per 100 g—over a threefold increase (Table 1). Co-administration with lysine-containing protein could theoretically influence lysine absorption. The higher arginine content may initially compete with lysine at the intestinal rBAT/b^0^,+AT transporter, though a distal gut transporter likely absorbs any remaining lysine. More probable, competition occurs during renal reabsorption via the same transporter, where plasma amino acid levels, rather than timing of intake, determine competition. This case report highlights the potential impact of GA1 medical formulas on lysine levels, which may offer protective benefits for patients at risk of striatal injury. However, careful evaluation is needed to prevent overprescribing these formulas particularly in patients with persistently low lysine levels who are otherwise meeting the DRI for protein.

**Table 1:** Nutrient summary report for well-baby formulas and GA1 infant formulas per 100 g powder.Ŧ

Name	Lysine (milligrams)	Arginine (milligrams)	Protein (grams)	Calories (kcal)
Similac Advance Powder (Abbott)	895	325	10.6	512
Enfamil Infant Powder (Mead Johnson)	910	300	10.1	510
Bobbie Organic Original Infant Powder (Bobbie)	960	350	10.4	520
GA-1 Anamix Early Years (Nutricia North America)	0	1180	13.5	473
Glutarex-2 (Abbott)	0	1550	15	480
GA Infant Formula (Mead Johnson)	0	1040	15.1	500

Ŧ Data provided by MetabolicPro [20] and manufacturer websites.

In conclusion, we observed an inverse relationship between the consumption of Lys-free, Trp-reduced, and Arg-fortified formula and blood lysine levels, independent of dietary protein intake from foods. We hypothesize that the high arginine content in GA1 medical formulas may interfere with lysine absorption in the intestine and/or reabsorption in the kidneys. Further research is needed to directly assess the impact of dietary arginine on plasma and urinary lysine levels, using quantifiable intake data and laboratory measurements, to refine dietary management strategies for patients with GA1.

## CRediT authorship contribution statement

**Grace Noh:** Writing – review & editing, Writing – original draft, Data curation, Conceptualization. **Jariya Upadia:** Writing – review & editing.

## Consent statement

Consent for publication was provided by the legal guardian.

## Ethics approval

Not required.

## Funding

The authors confirm independence from the sponsors.

## Declaration of competing interest

The authors have no competing interests to declare.

## Data Availability

The data that support the findings of this study are available from the corresponding author upon reasonable request.
